# Crystal structure of the catalytic C‐lobe of the HECT‐type ubiquitin ligase E6AP

**DOI:** 10.1002/pro.3832

**Published:** 2020-02-05

**Authors:** Lena K. Ries, Anna K. L. Liess, Christian G. Feiler, Donald E. Spratt, Edward D. Lowe, Sonja Lorenz

**Affiliations:** ^1^ Rudolf Virchow Center for Experimental Biomedicine University of Würzburg Würzburg Germany; ^2^ Gustaf H. Carlson School of Chemistry and Biochemistry, Clark University Worcester Massachusetts; ^3^ Department of Biochemistry Oxford University Oxford UK

**Keywords:** dimerization, domain swapping, E3 enzyme, UBE3A, X‐ray crystallography

## Abstract

The HECT‐type ubiquitin ligase E6AP (UBE3A) is critically involved in several neurodevelopmental disorders and human papilloma virus‐induced cervical tumorigenesis; the structural mechanisms underlying the activity of this crucial ligase, however, are incompletely understood. Here, we report a crystal structure of the C‐terminal lobe (“C‐lobe”) of the catalytic domain of E6AP that reveals two molecules in a domain‐swapped, dimeric arrangement. Interestingly, the molecular hinge that enables this structural reorganization with respect to the monomeric fold coincides with the active‐site region. While such dimerization is unlikely to occur in the context of full‐length E6AP, we noticed a similar domain swap in a crystal structure of the isolated C‐lobe of another HECT‐type ubiquitin ligase, HERC6. This may point to conformational strain in the active‐site region of HECT‐type ligases with possible implications for catalysis.

**Significance Statement:**

The HECT‐type ubiquitin ligase E6AP has key roles in human papilloma virus‐induced cervical tumorigenesis and certain neurodevelopmental disorders. Here, we present a crystal structure of the C‐terminal, catalytic lobe of E6AP, providing basic insight into the conformational properties of this functionally critical region of HECT‐type ligases.

## 1 INTRODUCTION

The ubiquitin system regulates protein functions at all levels, thereby orchestrating countless physiological and disease‐associated cellular pathways. Among the components of the ubiquitination machinery, ubiquitin ligases (E3 enzymes, E3s) are pivotal in determining the specificity of substrate selection and modification. E3s of the HECT (homologous to E6AP C‐terminus)‐subfamily share a C‐terminal catalytic domain that is comprised of two lobes—a ~30 kDa N‐lobe and a ~14 kDa C‐lobe—tethered by a flexible linker.^1^ Both lobes cooperate with each other in mediating sequential macromolecular interactions during ubiquitin transfer; in addition, the C‐lobe contains a catalytic cysteine that forms a thioester with the C‐terminus of ubiquitin in an intermediate step of catalysis. The specific recognition of substrates and regulatory factors by HECT‐type E3s is mediated by the diverse regions N‐terminal to the catalytic domain that are poorly characterized at a structural level.^2^

The founding member of the HECT E3 family, E6AP (UBE3A), has key roles in human diseases: its activity is hijacked by the E6 protein from high‐risk human papilloma viruses to promote the proteasomal degradation of the tumor suppressor p53, thereby driving cervical tumorigenesis; genetic up‐regulation of *E6AP* has been linked to autism spectrum disorders; and the deletion or down‐regulation of this ligase in the brain causes Angelman's syndrome.^3^ While the interactions of E6AP with ubiquitin have been analyzed and structural knowledge of its interplay with certain substrates is emerging,^4^, ^5^, ^6^, ^7^, ^8^ no specific inhibitors targeting this crucial ligase are available.^9^

Here, we report a crystal structure of the isolated C‐lobe of E6AP, revealing a three‐dimensionally domain‐swapped dimer. While distinct in structural detail, an overall similar crystallographic dimer is observed for the C‐lobe of HERC6 (PDB ID: http://firstglance.jmol.org/fg.htm?mol=5W87), suggesting that the conformational rearrangements with respect to the native, monomeric fold reflect dynamic properties of HECT‐type E3s beyond E6AP.

## 2 RESULTS AND DISCUSSION

**Figure 1:**
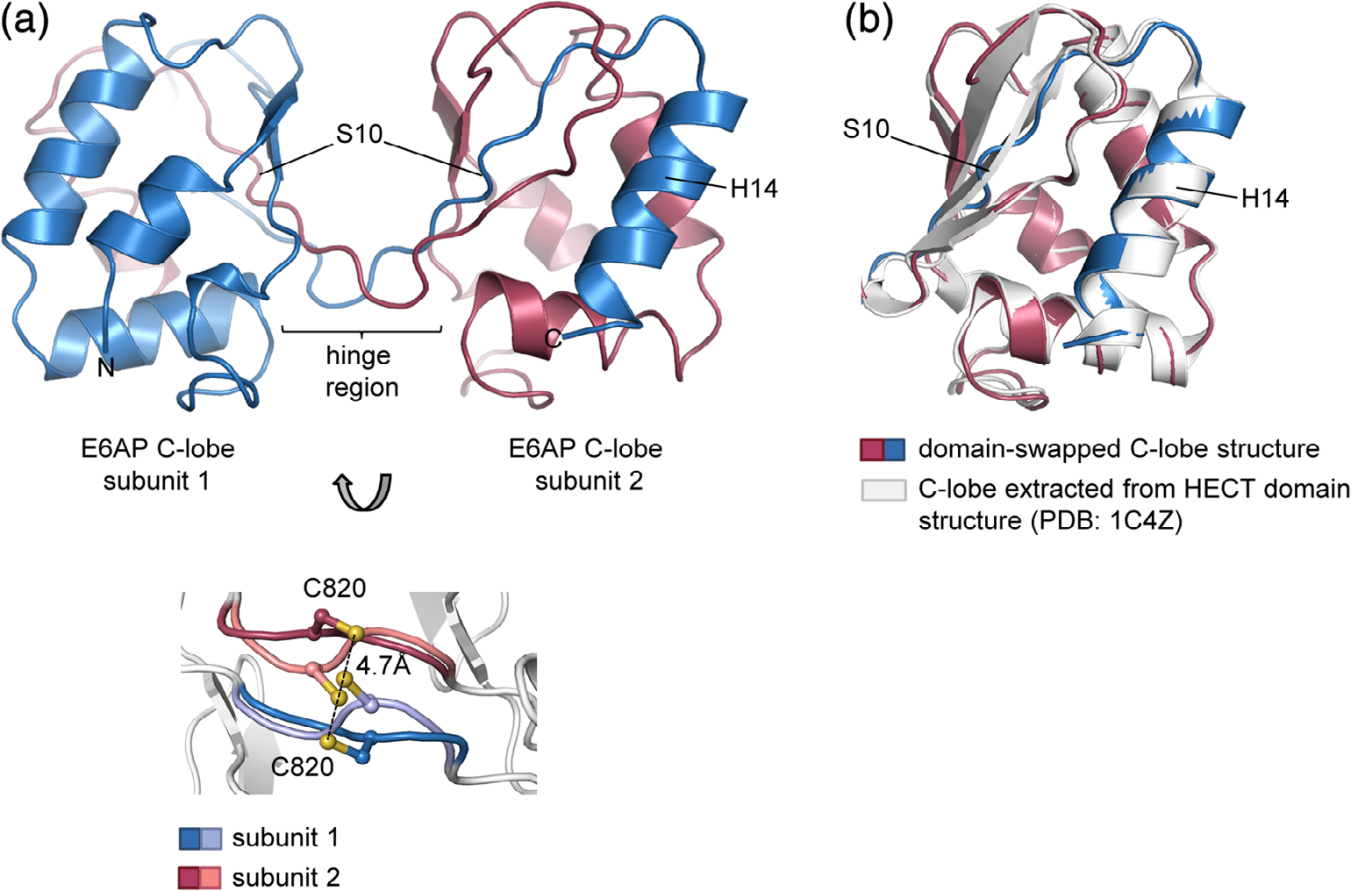
Crystal structure of a domain‐swapped E6AP C‐lobe dimer. (a) Crystal structure of the E6AP C‐lobe dimer determined in this study; the swapped secondary structure elements are labeled; only one conformation of the active‐site region is displayed (top). Detail of the active‐site region showing the two alternate conformations of residues 819–822 (colored) and the side chain of the catalytic Cys820 (bottom); the sulfur–sulfur distance of the non‐disulfide bonded state is indicated. (b) Superposition of one domain‐swapped unit extracted from (a) with the crystal structure of the (monomeric) C‐lobe, extracted from a structure of the E6AP HECT domain (PDB ID: http://firstglance.jmol.org/fg.htm?mol=1C4Z 1; C_α_‐RMSD: 0.48 Å)

We determined a crystal structure of the C‐lobe of E6AP at 1.3 Å resolution (Table 1). The structure reveals a dimeric arrangement, in which the C‐terminal α‐helix (H14) and adjacent β‐strand (S10) of two symmetry‐related molecules have undergone three‐dimensional domain swapping (Fig. 1a, top panel). This term describes a phenomenon where identical proteins exchange part of their structure to give rise to an oligomer, of which individual subunits have a similar fold as an isolated monomer.^10^ Each composite subunit of the crystallographic C‐lobe dimer thus recapitulates the canonical, globular α/β‐fold seen in the context of the HECT domain of E6AP (RMSD of 0.48 Å in 104 equivalent C_α_‐positions with respect to the C‐lobe structure extracted from PDB ID: http://firstglance.jmol.org/fg.htm?mol=1C4Z, chain A)^1^ (Fig. 1b). Interestingly, the hinge region that mediates the domain swap coincides with the active‐site region (Thr819, Cys820, Phe821, and Asn822) and adopts two conformations (Fig. 1a, bottom panel). As a consequence, the catalytic cysteine (Cys820) residues of the two subunits are either in immediate proximity or at a sulfur–sulfur distance of 4.7 Å, which we interpret as a mixture of disulfide‐bonded and reduced states. We speculate that these alternative oxidation states originate from radiation‐induced, partial cleavage of an intermolecular disulfide bond formed during the crystallization process.

**Table 1:** X‐ray crystallographic data collection and refinement statistics

**Data collection**	
Wavelength	0.9184
Space group	I 4 2 2
Unit cell parameters	
*a*, *b*, *c* (Å)	72.28, 72.28, 98.97
*α*, *β*, *γ* (°)	90, 90, 90
Resolution (Å)	30.03–1.30 (1.35–1.30)
Total reflections	65,057 (6,414)
Unique reflections	32,542 (3,211)
*R*_pim_	0.01279 (0.5023)
CC1/2	1 (0.676)
Mean *I*/*σ*(*I*)	26.56 (1.49)
Completeness (%)	99.92 (99.63)
Multiplicity	2.0 (2.0)
Wilson B‐factor	17.13
**Refinement**	
*R*_work_	17.8 (28.8)
*R*_free_	20.7 (29.8)
No. of non‐hydrogen atoms	1,051
Protein	954
Ligand	9
Water	88
Average B‐factors	23.57
Protein	22.79
Ligands	30.43
Water	31.35
RMS deviations from ideality	
Bond lengths (Å)	0.014
Bond angles (°)	1.9
Ramachandran statistics	
Favored (%)	97.09
Disallowed (%)	0.0
MolProbity clash score	3.09
MolProbity overall score	1.18

*Note*: Values in parentheses correspond to the highest‐resolution shell. Data were collected from a single crystal.

We and others have previously reported that the C‐lobe and the HECT domain of E6AP are monomeric in solution,^1^, ^4^ consistent with cell‐based studies on the full‐length ligase.^11^, ^12^ Yet, evidence for a trimeric state in vitro and oligomerization of E6AP in the cell has also been presented.^6^, ^13^, ^14^, ^15^, ^16^ Either way, we posit that the observed domain swapping specifically occurs in the context of the isolated C‐lobe and is unlikely to happen in the presence of the N‐lobe. In line with this notion, the C‐lobe dimer is incompatible with the position of the N‐lobe in several alternative conformations in which HECT domains have been crystallized, including an “L”‐shaped state (PDB ID: http://firstglance.jmol.org/fg.htm?mol=1C4Z 1) and a “T”‐shaped complex with an E2 (UBCH7), as required for the trans‐thioesterification reaction (analogous to PDB ID: http://firstglance.jmol.org/fg.htm?mol=3JVZ 17).

**Figure 2:**
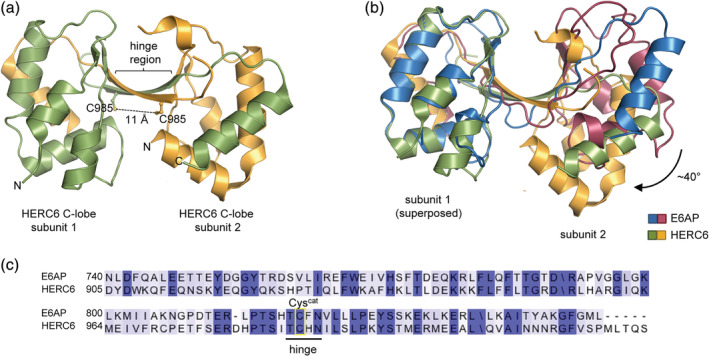
Comparison of the crystallographic, domain‐swapped dimers of the C‐lobe of E6AP and HERC6. (a) Crystal structure of a domain‐swapped dimer of the C‐lobe of HERC6 (PDB ID: http://firstglance.jmol.org/fg.htm?mol=5W87). The side chain of the catalytic Cys985 is shown for each molecule, along with the sulfur–sulfur distance (b) Superposition of the domain‐swapped, dimeric structures of the C‐lobe of E6AP and HERC6. (c) Sequence alignment for the C‐lobes of E6AP and HERC6 (rendered with Jalview^18^ and colored according to conservation), highlighting the hinge region and the catalytic cysteine (Cys^cat^)

Interestingly, a similar domain‐swapped, crystallographic dimer is formed by the isolated C‐lobe of another HECT‐type E3 HERC6 (Fig. 2b), which may present a bona fide ubiquitin‐directed ligase in the human system (while its murine orthologue is specific for the ubiquitin‐like modifier ISG15^19^, ^20^). As for E6AP, the hinge region in HERC6 comprises the active site, yet the swapped region is slightly offset and the subunits are tilted by ~40° with respect to the E6AP C‐lobe dimer, thus positioning the catalytic cysteine residues of the two subunits at a distance of 11 Å (Fig. 2a,b). This comparison implies that domain swapping can occur in the isolated C‐lobes of HECT‐type E3s beyond E6AP and independently of intermolecular disulfide formation.

While its biological relevance needs to be evaluated on a case‐by‐case basis, domain swapping typically reports on conformational tension and can thus unveil energetic properties of a monomeric structure that are hard to prove otherwise.^21^ We assume that the domain‐swapped dimers observed for the C‐lobes of HECT‐type E3s represent an artifact of extracting this region from the native protein context. At the same time, the structural reorganization may indicate inherent conformational strain in the hinge region within the monomeric fold. In line with this notion, the hinge region is the shortest among the loops connecting individual β‐strands of the C‐lobe and may thus resemble a loaded spring that releases conformational tension upon swapping.^21^ In the context of the full‐length protein—where we expect domain swapping is inhibited—the conformational tension in the active‐site region may affect the energetics and dynamic behavior of the entire C‐lobe. Further studies will be required to explore this hypothesis and interrogate whether these “hidden” conformational properties affect catalysis.

## 3 MATERIALS AND METHODS

### 3.1 *Protein preparation and crystallization*

The nucleotide sequence encoding the C‐lobe (residues 741‐852; numbering according to isoform 1) of E6AP was cloned into a pET‐28a vector (Merck), modified to encode an N‐terminal 3C protease‐cleavable His_6_‐tag. The protein was recombinantly expressed in *E. coli* BL21 (DE3) and purified as previously described.^4^ After removal of the His_6_‐tag, the protein was subjected to size‐exclusion chromatography (Superdex HiLoad 26/600 75 pg column, GE Healthcare, Uppsala, Sweden) in 75 mM Tris, pH 7.5, 200 mM NaCl. The purified protein crystallized at concentrations ≥30 mg/ml and 20°C in sitting drops containing 0.1 M calcium chloride, 0.1 M sodium acetate pH 4.6, and 30% PEG400; the particular crystal used for structure determination was obtained at 60 mg/ml protein concentration; no additional cryo‐protection was required.

### 3.2 *X‐ray crystallographic data collection and structure determination*

Diffraction data were collected at 100 K, beamline BL14.1 at the BESSY II, Helmholtz‐Zentrum Berlin and processed with XDS.^22^ Molecular replacement was performed with Phaser,^23^ as implemented in the collaborative computational project no. 4 (ccp4) suite (RRID: SCR_007255),^24^ using a structure of the E6AP C‐lobe as a search model (extracted from PDB ID: http://firstglance.jmol.org/fg.htm?mol=1C4Z 1); refinement with Phenix (RRID: SCR_014224)^25^ and REFMAC5 (RRID: SCR_014225)^26^ with individual B‐factors and TLS (translation/libration/screw); and model building with Coot (RRID: SCR_014222).^27^

## 4 ACCESSION NUMBERS

Atomic coordinates and structure factors have been deposited in the PDB under accession code http://firstglance.jmol.org/fg.htm?mol=6TGK.

## CONFLICT OF INTEREST

The authors declare no potential conflict of interest.
